# Supplementary material for: Modeling glioblastoma heterogeneity as a dynamic network of cell states
Source: Mol Syst Biol. 2021 Sep 16;17(9):e10105. doi: 10.15252/msb.202010105 (PMC8444284; doi:10.15252/msb.202010105)
Supplement: Supplementary file 6 — Source Data for Figure 5 [file MSB-17-e10105-s004.zip › Figure5A_sourcedata/GSEA_3017/nosigdb_stateB.GseaPreranked.1622795021725/RICHARDS_NOWAKOWSKI_SCIENCE_2017_IPC_DIV2_UPREG.html]

Details for gene set RICHARDS\_NOWAKOWSKI\_SCIENCE\_2017\_IPC\_DIV2\_UPREG[GSEA]

|  || Dataset | state43017 |
| Phenotype | NoPhenotypeAvailable |
| Upregulated in class | na\_pos |
| GeneSet | RICHARDS\_NOWAKOWSKI\_SCIENCE\_2017\_IPC\_DIV2\_UPREG |
| Enrichment Score (ES) | 0.50182223 |
| Normalized Enrichment Score (NES) | 3.2607148 |
| Nominal p-value | 0.0 |
| FDR q-value | 0.0 |
| FWER p-Value | 0.0 |
Table: GSEA Results Summary

  

Fig 1: Enrichment plot: RICHARDS\_NOWAKOWSKI\_SCIENCE\_2017\_IPC\_DIV2\_UPREG      
 Profile of the Running ES Score & Positions of GeneSet Members on the Rank Ordered List

  

| PROBE | GENE SYMBOL | GENE\_TITLE | RANK IN GENE LIST | RANK METRIC SCORE | RUNNING ES | CORE ENRICHMENT || 1 | HIST1H4C |  |  | 0 | 1.373 | 0.0101 | Yes |
| 2 | HIST1H1B |  |  | 1 | 1.272 | 0.0194 | Yes |
| 3 | HIST1H1D |  |  | 2 | 1.180 | 0.0281 | Yes |
| 4 | TOP2A |  |  | 3 | 1.079 | 0.0360 | Yes |
| 5 | HIST1H1C |  |  | 4 | 1.041 | 0.0436 | Yes |
| 6 | HIST1H1E |  |  | 5 | 0.983 | 0.0508 | Yes |
| 7 | RRM2 |  |  | 7 | 0.875 | 0.0551 | Yes |
| 8 | FAM111A |  |  | 10 | 0.813 | 0.0566 | Yes |
| 9 | UBE2C |  |  | 11 | 0.802 | 0.0625 | Yes |
| 10 | TPX2 |  |  | 12 | 0.801 | 0.0684 | Yes |
| 11 | NUSAP1 |  |  | 13 | 0.801 | 0.0743 | Yes |
| 12 | HMGB2 |  |  | 14 | 0.786 | 0.0801 | Yes |
| 13 | FBXO5 |  |  | 15 | 0.785 | 0.0858 | Yes |
| 14 | NCAPG |  |  | 16 | 0.778 | 0.0915 | Yes |
| 15 | SPC25 |  |  | 17 | 0.763 | 0.0971 | Yes |
| 16 | PRC1 |  |  | 18 | 0.756 | 0.1027 | Yes |
| 17 | CDCA2 |  |  | 19 | 0.751 | 0.1082 | Yes |
| 18 | ESCO2 |  |  | 20 | 0.742 | 0.1136 | Yes |
| 19 | TACC3 |  |  | 21 | 0.741 | 0.1191 | Yes |
| 20 | GTSE1 |  |  | 22 | 0.732 | 0.1244 | Yes |
| 21 | HJURP |  |  | 23 | 0.731 | 0.1298 | Yes |
| 22 | KIF23 |  |  | 24 | 0.721 | 0.1351 | Yes |
| 23 | ASPM |  |  | 26 | 0.695 | 0.1380 | Yes |
| 24 | NDC80 |  |  | 27 | 0.695 | 0.1431 | Yes |
| 25 | ATAD2 |  |  | 28 | 0.685 | 0.1481 | Yes |
| 26 | KIF15 |  |  | 29 | 0.684 | 0.1531 | Yes |
| 27 | ARHGAP11A |  |  | 30 | 0.683 | 0.1582 | Yes |
| 28 | KPNA2 |  |  | 33 | 0.676 | 0.1587 | Yes |
| 29 | CENPF |  |  | 34 | 0.670 | 0.1637 | Yes |
| 30 | KIF4A |  |  | 37 | 0.662 | 0.1641 | Yes |
| 31 | ANLN |  |  | 39 | 0.659 | 0.1668 | Yes |
| 32 | RAD51AP1 |  |  | 40 | 0.657 | 0.1716 | Yes |
| 33 | SMC4 |  |  | 41 | 0.655 | 0.1764 | Yes |
| 34 | MELK |  |  | 42 | 0.655 | 0.1812 | Yes |
| 35 | CENPE |  |  | 43 | 0.654 | 0.1860 | Yes |
| 36 | NUF2 |  |  | 44 | 0.651 | 0.1908 | Yes |
| 37 | PRR11 |  |  | 45 | 0.649 | 0.1955 | Yes |
| 38 | DLGAP5 |  |  | 46 | 0.647 | 0.2003 | Yes |
| 39 | KIFC1 |  |  | 47 | 0.645 | 0.2050 | Yes |
| 40 | KIF18A |  |  | 48 | 0.639 | 0.2097 | Yes |
| 41 | DHFR |  |  | 49 | 0.638 | 0.2144 | Yes |
| 42 | NCAPG2 |  |  | 50 | 0.636 | 0.2191 | Yes |
| 43 | CDCA5 |  |  | 52 | 0.632 | 0.2215 | Yes |
| 44 | CKAP2 |  |  | 53 | 0.628 | 0.2261 | Yes |
| 45 | CCNA2 |  |  | 54 | 0.626 | 0.2307 | Yes |
| 46 | FANCI |  |  | 56 | 0.615 | 0.2330 | Yes |
| 47 | MYBL2 |  |  | 58 | 0.612 | 0.2353 | Yes |
| 48 | CKAP2L |  |  | 59 | 0.611 | 0.2398 | Yes |
| 49 | CENPU |  |  | 60 | 0.605 | 0.2443 | Yes |
| 50 | CENPK |  |  | 62 | 0.603 | 0.2465 | Yes |
| 51 | TYMS |  |  | 63 | 0.601 | 0.2509 | Yes |
| 52 | BUB1B |  |  | 64 | 0.596 | 0.2553 | Yes |
| 53 | CCDC34 |  |  | 65 | 0.595 | 0.2596 | Yes |
| 54 | KIF2C |  |  | 66 | 0.591 | 0.2640 | Yes |
| 55 | SMC2 |  |  | 68 | 0.586 | 0.2661 | Yes |
| 56 | CDK1 |  |  | 69 | 0.585 | 0.2704 | Yes |
| 57 | H2AFX |  |  | 71 | 0.576 | 0.2724 | Yes |
| 58 | CKS1B |  |  | 72 | 0.575 | 0.2766 | Yes |
| 59 | RACGAP1 |  |  | 75 | 0.572 | 0.2764 | Yes |
| 60 | FOXM1 |  |  | 76 | 0.570 | 0.2806 | Yes |
| 61 | MAD2L1 |  |  | 77 | 0.569 | 0.2848 | Yes |
| 62 | PLK1 |  |  | 78 | 0.567 | 0.2889 | Yes |
| 63 | DBF4 |  |  | 79 | 0.567 | 0.2931 | Yes |
| 64 | CDCA8 |  |  | 82 | 0.562 | 0.2928 | Yes |
| 65 | CKS2 |  |  | 83 | 0.558 | 0.2969 | Yes |
| 66 | ECT2 |  |  | 84 | 0.558 | 0.3010 | Yes |
| 67 | KIF14 |  |  | 85 | 0.557 | 0.3051 | Yes |
| 68 | E2F7 |  |  | 86 | 0.556 | 0.3092 | Yes |
| 69 | AURKB |  |  | 89 | 0.548 | 0.3088 | Yes |
| 70 | SPC24 |  |  | 91 | 0.544 | 0.3106 | Yes |
| 71 | CENPM |  |  | 92 | 0.544 | 0.3146 | Yes |
| 72 | NCAPH |  |  | 94 | 0.543 | 0.3164 | Yes |
| 73 | PBK |  |  | 95 | 0.540 | 0.3204 | Yes |
| 74 | CSRP2 |  |  | 96 | 0.539 | 0.3243 | Yes |
| 75 | XRCC2 |  |  | 98 | 0.530 | 0.3260 | Yes |
| 76 | SKA3 |  |  | 99 | 0.525 | 0.3299 | Yes |
| 77 | TMEM106C |  |  | 100 | 0.523 | 0.3337 | Yes |
| 78 | RRM1 |  |  | 103 | 0.520 | 0.3332 | Yes |
| 79 | CMC2 |  |  | 105 | 0.516 | 0.3348 | Yes |
| 80 | CCNF |  |  | 106 | 0.513 | 0.3385 | Yes |
| 81 | SDC2 |  |  | 107 | 0.513 | 0.3423 | Yes |
| 82 | CDKN3 |  |  | 108 | 0.513 | 0.3461 | Yes |
| 83 | CEP135 |  |  | 109 | 0.513 | 0.3498 | Yes |
| 84 | UBE2T |  |  | 110 | 0.512 | 0.3536 | Yes |
| 85 | CDCA3 |  |  | 111 | 0.511 | 0.3573 | Yes |
| 86 | DIAPH3 |  |  | 112 | 0.510 | 0.3611 | Yes |
| 87 | BUB1 |  |  | 113 | 0.508 | 0.3648 | Yes |
| 88 | RAD21 |  |  | 115 | 0.506 | 0.3663 | Yes |
| 89 | BIRC5 |  |  | 116 | 0.504 | 0.3700 | Yes |
| 90 | CLSPN |  |  | 117 | 0.503 | 0.3737 | Yes |
| 91 | DTYMK |  |  | 118 | 0.503 | 0.3774 | Yes |
| 92 | CEP152 |  |  | 119 | 0.502 | 0.3811 | Yes |
| 93 | ASF1B |  |  | 120 | 0.501 | 0.3848 | Yes |
| 94 | PSMC3IP |  |  | 122 | 0.495 | 0.3862 | Yes |
| 95 | KIF18B |  |  | 123 | 0.494 | 0.3898 | Yes |
| 96 | DUT |  |  | 124 | 0.494 | 0.3934 | Yes |
| 97 | E2F8 |  |  | 125 | 0.493 | 0.3971 | Yes |
| 98 | TMPO |  |  | 127 | 0.492 | 0.3985 | Yes |
| 99 | ITGB3BP |  |  | 128 | 0.490 | 0.4021 | Yes |
| 100 | LMNB2 |  |  | 129 | 0.488 | 0.4056 | Yes |
| 101 | SKA1 |  |  | 131 | 0.487 | 0.4070 | Yes |
| 102 | SHCBP1 |  |  | 133 | 0.484 | 0.4084 | Yes |
| 103 | GMNN |  |  | 135 | 0.484 | 0.4097 | Yes |
| 104 | NCAPD2 |  |  | 136 | 0.483 | 0.4133 | Yes |
| 105 | TTK |  |  | 137 | 0.482 | 0.4168 | Yes |
| 106 | FANCB |  |  | 138 | 0.478 | 0.4203 | Yes |
| 107 | INCENP |  |  | 140 | 0.476 | 0.4216 | Yes |
| 108 | C21orf58 |  |  | 142 | 0.470 | 0.4229 | Yes |
| 109 | CDCA4 |  |  | 148 | 0.461 | 0.4153 | Yes |
| 110 | FANCD2 |  |  | 149 | 0.461 | 0.4187 | Yes |
| 111 | ORC6 |  |  | 150 | 0.461 | 0.4221 | Yes |
| 112 | KIF22 |  |  | 152 | 0.459 | 0.4233 | Yes |
| 113 | NCAPD3 |  |  | 153 | 0.458 | 0.4266 | Yes |
| 114 | CDC45 |  |  | 154 | 0.456 | 0.4300 | Yes |
| 115 | SPAG5 |  |  | 156 | 0.455 | 0.4311 | Yes |
| 116 | MCM8 |  |  | 158 | 0.454 | 0.4323 | Yes |
| 117 | GPC6 |  |  | 159 | 0.454 | 0.4356 | Yes |
| 118 | CEP78 |  |  | 161 | 0.451 | 0.4367 | Yes |
| 119 | POLQ |  |  | 162 | 0.450 | 0.4400 | Yes |
| 120 | CENPQ |  |  | 163 | 0.446 | 0.4433 | Yes |
| 121 | RPA1 |  |  | 165 | 0.445 | 0.4443 | Yes |
| 122 | TK1 |  |  | 167 | 0.444 | 0.4454 | Yes |
| 123 | BRIP1 |  |  | 168 | 0.443 | 0.4487 | Yes |
| 124 | ATAD5 |  |  | 170 | 0.442 | 0.4497 | Yes |
| 125 | BRCA1 |  |  | 171 | 0.440 | 0.4529 | Yes |
| 126 | CDK5RAP2 |  |  | 173 | 0.438 | 0.4540 | Yes |
| 127 | BRCA2 |  |  | 174 | 0.436 | 0.4572 | Yes |
| 128 | RBBP8 |  |  | 176 | 0.436 | 0.4582 | Yes |
| 129 | DEPDC1B |  |  | 177 | 0.436 | 0.4614 | Yes |
| 130 | MKI67 |  |  | 179 | 0.436 | 0.4624 | Yes |
| 131 | FEN1 |  |  | 180 | 0.435 | 0.4656 | Yes |
| 132 | RHNO1 |  |  | 183 | 0.432 | 0.4643 | Yes |
| 133 | USP1 |  |  | 185 | 0.430 | 0.4653 | Yes |
| 134 | MIS18BP1 |  |  | 186 | 0.430 | 0.4685 | Yes |
| 135 | MGME1 |  |  | 188 | 0.428 | 0.4694 | Yes |
| 136 | BARD1 |  |  | 189 | 0.427 | 0.4725 | Yes |
| 137 | MND1 |  |  | 193 | 0.424 | 0.4691 | Yes |
| 138 | NEK2 |  |  | 194 | 0.423 | 0.4722 | Yes |
| 139 | NEDD1 |  |  | 195 | 0.423 | 0.4753 | Yes |
| 140 | MIS18A |  |  | 196 | 0.421 | 0.4784 | Yes |
| 141 | MCM4 |  |  | 199 | 0.418 | 0.4770 | Yes |
| 142 | DSN1 |  |  | 202 | 0.413 | 0.4757 | Yes |
| 143 | SYNE2 |  |  | 203 | 0.413 | 0.4787 | Yes |
| 144 | CCP110 |  |  | 204 | 0.411 | 0.4817 | Yes |
| 145 | ZWINT |  |  | 212 | 0.407 | 0.4694 | Yes |
| 146 | LIG1 |  |  | 214 | 0.407 | 0.4702 | Yes |
| 147 | CCNE2 |  |  | 215 | 0.406 | 0.4731 | Yes |
| 148 | H2AFZ |  |  | 216 | 0.405 | 0.4761 | Yes |
| 149 | MNS1 |  |  | 219 | 0.401 | 0.4747 | Yes |
| 150 | SSX2IP |  |  | 222 | 0.399 | 0.4732 | Yes |
| 151 | CENPO |  |  | 223 | 0.398 | 0.4761 | Yes |
| 152 | FANCA |  |  | 227 | 0.395 | 0.4724 | Yes |
| 153 | TIMELESS |  |  | 228 | 0.395 | 0.4753 | Yes |
| 154 | STIL |  |  | 231 | 0.393 | 0.4738 | Yes |
| 155 | EZH2 |  |  | 236 | 0.388 | 0.4679 | Yes |
| 156 | VRK1 |  |  | 238 | 0.388 | 0.4686 | Yes |
| 157 | CDC6 |  |  | 239 | 0.388 | 0.4714 | Yes |
| 158 | PARPBP |  |  | 240 | 0.387 | 0.4743 | Yes |
| 159 | PCNA |  |  | 242 | 0.386 | 0.4749 | Yes |
| 160 | HMGN2 |  |  | 243 | 0.386 | 0.4777 | Yes |
| 161 | SUGP2 |  |  | 244 | 0.385 | 0.4806 | Yes |
| 162 | POLA1 |  |  | 247 | 0.383 | 0.4790 | Yes |
| 163 | RTKN2 |  |  | 248 | 0.379 | 0.4818 | Yes |
| 164 | CCNB2 |  |  | 250 | 0.379 | 0.4824 | Yes |
| 165 | POLD3 |  |  | 252 | 0.377 | 0.4829 | Yes |
| 166 | MPHOSPH9 |  |  | 254 | 0.375 | 0.4835 | Yes |
| 167 | PLK4 |  |  | 255 | 0.374 | 0.4862 | Yes |
| 168 | RAD18 |  |  | 256 | 0.373 | 0.4890 | Yes |
| 169 | RAD23A |  |  | 258 | 0.371 | 0.4895 | Yes |
| 170 | C18orf54 |  |  | 259 | 0.371 | 0.4922 | Yes |
| 171 | RFC5 |  |  | 260 | 0.370 | 0.4949 | Yes |
| 172 | CENPJ |  |  | 261 | 0.370 | 0.4977 | Yes |
| 173 | TUBB |  |  | 263 | 0.368 | 0.4982 | Yes |
| 174 | ASRGL1 |  |  | 266 | 0.366 | 0.4965 | Yes |
| 175 | CENPW |  |  | 267 | 0.366 | 0.4991 | Yes |
| 176 | TUBG1 |  |  | 268 | 0.365 | 0.5018 | Yes |
| 177 | SMC1A |  |  | 274 | 0.361 | 0.4935 | No |
| 178 | PCNT |  |  | 275 | 0.360 | 0.4961 | No |
| 179 | CHAF1A |  |  | 276 | 0.359 | 0.4988 | No |
| 180 | MMS22L |  |  | 277 | 0.359 | 0.5014 | No |
| 181 | ANP32E |  |  | 280 | 0.358 | 0.4997 | No |
| 182 | GINS1 |  |  | 283 | 0.357 | 0.4979 | No |
| 183 | HAUS8 |  |  | 293 | 0.353 | 0.4808 | No |
| 184 | RFC4 |  |  | 295 | 0.351 | 0.4811 | No |
| 185 | SUZ12 |  |  | 297 | 0.349 | 0.4815 | No |
| 186 | DTL |  |  | 305 | 0.345 | 0.4687 | No |
| 187 | PRKDC |  |  | 306 | 0.344 | 0.4712 | No |
| 188 | CDC25C |  |  | 307 | 0.343 | 0.4737 | No |
| 189 | CENPN |  |  | 309 | 0.343 | 0.4741 | No |
| 190 | PKP4 |  |  | 311 | 0.342 | 0.4744 | No |
| 191 | FAM111B |  |  | 314 | 0.342 | 0.4725 | No |
| 192 | DEK |  |  | 316 | 0.341 | 0.4728 | No |
| 193 | PHF19 |  |  | 319 | 0.339 | 0.4709 | No |
| 194 | CSE1L |  |  | 321 | 0.338 | 0.4712 | No |
| 195 | RFC3 |  |  | 322 | 0.338 | 0.4737 | No |
| 196 | CIT |  |  | 326 | 0.336 | 0.4696 | No |
| 197 | RFC2 |  |  | 331 | 0.333 | 0.4632 | No |
| 198 | HIRIP3 |  |  | 336 | 0.331 | 0.4569 | No |
| 199 | BTG3 |  |  | 337 | 0.331 | 0.4593 | No |
| 200 | UBR7 |  |  | 340 | 0.330 | 0.4573 | No |
| 201 | NFATC2IP |  |  | 343 | 0.328 | 0.4554 | No |
| 202 | PHGDH |  |  | 344 | 0.328 | 0.4578 | No |
| 203 | LRRCC1 |  |  | 349 | 0.326 | 0.4514 | No |
| 204 | HAT1 |  |  | 352 | 0.324 | 0.4494 | No |
| 205 | TMEM237 |  |  | 353 | 0.324 | 0.4518 | No |
| 206 | ARHGAP11B |  |  | 356 | 0.323 | 0.4497 | No |
| 207 | CCDC14 |  |  | 357 | 0.322 | 0.4521 | No |
| 208 | LMNB1 |  |  | 363 | 0.321 | 0.4435 | No |
| 209 | XPO1 |  |  | 365 | 0.321 | 0.4437 | No |
| 210 | WDR34 |  |  | 366 | 0.321 | 0.4460 | No |
| 211 | RBBP7 |  |  | 367 | 0.321 | 0.4484 | No |
| 212 | PARP2 |  |  | 372 | 0.319 | 0.4419 | No |
| 213 | SOGA1 |  |  | 378 | 0.314 | 0.4333 | No |
| 214 | PIN1 |  |  | 379 | 0.314 | 0.4356 | No |
| 215 | CEP128 |  |  | 381 | 0.314 | 0.4357 | No |
| 216 | ZNF714 |  |  | 382 | 0.313 | 0.4380 | No |
| 217 | GSTCD |  |  | 384 | 0.312 | 0.4381 | No |
| 218 | PRIM2 |  |  | 391 | 0.311 | 0.4272 | No |
| 219 | KIF20A |  |  | 392 | 0.311 | 0.4295 | No |
| 220 | HAUS2 |  |  | 393 | 0.311 | 0.4318 | No |
| 221 | CEP192 |  |  | 395 | 0.310 | 0.4319 | No |
| 222 | EZR |  |  | 403 | 0.307 | 0.4188 | No |
| 223 | RHEB |  |  | 404 | 0.307 | 0.4210 | No |
| 224 | BLM |  |  | 408 | 0.305 | 0.4167 | No |
| 225 | CENPC |  |  | 409 | 0.304 | 0.4189 | No |
| 226 | LRR1 |  |  | 410 | 0.304 | 0.4211 | No |
| 227 | PTTG1 |  |  | 413 | 0.302 | 0.4190 | No |
| 228 | ZGRF1 |  |  | 415 | 0.302 | 0.4190 | No |
| 229 | CD47 |  |  | 419 | 0.300 | 0.4146 | No |
| 230 | DSCC1 |  |  | 422 | 0.299 | 0.4124 | No |
| 231 | PPME1 |  |  | 423 | 0.299 | 0.4146 | No |
| 232 | AHI1 |  |  | 425 | 0.299 | 0.4146 | No |
| 233 | NUP107 |  |  | 430 | 0.298 | 0.4080 | No |
| 234 | TMEM47 |  |  | 437 | 0.295 | 0.3970 | No |
| 235 | GINM1 |  |  | 439 | 0.295 | 0.3970 | No |
| 236 | PHIP |  |  | 440 | 0.295 | 0.3992 | No |
| 237 | IQGAP3 |  |  | 447 | 0.294 | 0.3882 | No |
| 238 | TEX30 |  |  | 448 | 0.294 | 0.3903 | No |
| 239 | CCDC150 |  |  | 449 | 0.293 | 0.3925 | No |
| 240 | MZT1 |  |  | 456 | 0.291 | 0.3814 | No |
| 241 | CBX5 |  |  | 457 | 0.291 | 0.3836 | No |
| 242 | SAE1 |  |  | 463 | 0.289 | 0.3747 | No |
| 243 | RFWD3 |  |  | 464 | 0.289 | 0.3769 | No |
| 244 | NUP155 |  |  | 468 | 0.288 | 0.3724 | No |
| 245 | NUP50 |  |  | 470 | 0.287 | 0.3723 | No |
| 246 | YEATS4 |  |  | 476 | 0.286 | 0.3634 | No |
| 247 | CHEK1 |  |  | 477 | 0.286 | 0.3655 | No |
| 248 | RNASEH2A |  |  | 478 | 0.285 | 0.3676 | No |
| 249 | ARHGAP33 |  |  | 482 | 0.285 | 0.3631 | No |
| 250 | NUCKS1 |  |  | 483 | 0.285 | 0.3652 | No |
| 251 | HADH |  |  | 485 | 0.284 | 0.3651 | No |
| 252 | HMGXB4 |  |  | 491 | 0.282 | 0.3562 | No |
| 253 | CENPH |  |  | 501 | 0.281 | 0.3385 | No |
| 254 | PSMC3 |  |  | 506 | 0.280 | 0.3318 | No |
| 255 | WDHD1 |  |  | 513 | 0.278 | 0.3207 | No |
| 256 | TTF2 |  |  | 514 | 0.278 | 0.3228 | No |
| 257 | EML1 |  |  | 515 | 0.278 | 0.3248 | No |
| 258 | LSM3 |  |  | 522 | 0.277 | 0.3137 | No |
| 259 | DNMT1 |  |  | 523 | 0.277 | 0.3157 | No |
| 260 | NFIC |  |  | 531 | 0.276 | 0.3024 | No |
| 261 | NUCB2 |  |  | 533 | 0.275 | 0.3022 | No |
| 262 | HIST1H2AJ |  |  | 541 | 0.273 | 0.2889 | No |
| 263 | FIGNL1 |  |  | 542 | 0.273 | 0.2909 | No |
| 264 | ALYREF |  |  | 546 | 0.273 | 0.2863 | No |
| 265 | CALU |  |  | 552 | 0.272 | 0.2773 | No |
| 266 | NUP85 |  |  | 563 | 0.270 | 0.2574 | No |
| 267 | GGH |  |  | 564 | 0.270 | 0.2594 | No |
| 268 | CDK2 |  |  | 565 | 0.270 | 0.2613 | No |
| 269 | DDX11 |  |  | 568 | 0.269 | 0.2589 | No |
| 270 | CTNNAL1 |  |  | 570 | 0.269 | 0.2587 | No |
| 271 | NUP205 |  |  | 571 | 0.269 | 0.2607 | No |
| 272 | CNTRL |  |  | 575 | 0.269 | 0.2561 | No |
| 273 | RPA3 |  |  | 584 | 0.267 | 0.2405 | No |
| 274 | ZNF682 |  |  | 585 | 0.267 | 0.2425 | No |
| 275 | ZNF850 |  |  | 589 | 0.266 | 0.2378 | No |
| 276 | SLC43A3 |  |  | 590 | 0.265 | 0.2398 | No |
| 277 | KPNB1 |  |  | 600 | 0.263 | 0.2220 | No |
| 278 | ERI1 |  |  | 604 | 0.262 | 0.2173 | No |
| 279 | KDELC2 |  |  | 612 | 0.261 | 0.2039 | No |
| 280 | TBC1D31 |  |  | 616 | 0.261 | 0.1992 | No |
| 281 | PAX6 |  |  | 617 | 0.261 | 0.2011 | No |
| 282 | NDE1 |  |  | 623 | 0.259 | 0.1921 | No |
| 283 | UBA2 |  |  | 626 | 0.259 | 0.1896 | No |
| 284 | CHCHD3 |  |  | 628 | 0.259 | 0.1893 | No |
| 285 | ARL6IP6 |  |  | 629 | 0.258 | 0.1912 | No |
| 286 | CENPI |  |  | 630 | 0.258 | 0.1931 | No |
| 287 | HNRNPUL1 |  |  | 637 | 0.257 | 0.1818 | No |
| 288 | HEG1 |  |  | 639 | 0.257 | 0.1815 | No |
| 289 | EXOSC8 |  |  | 647 | 0.256 | 0.1680 | No |
| 290 | UHRF1 |  |  | 652 | 0.255 | 0.1611 | No |
| 291 | RDX |  |  | 658 | 0.255 | 0.1520 | No |
| 292 | PCBP2 |  |  | 660 | 0.255 | 0.1517 | No |
| 293 | GPAA1 |  |  | 665 | 0.253 | 0.1448 | No |
| 294 | SPATA5 |  |  | 675 | 0.252 | 0.1269 | No |
| 295 | ITGB1 |  |  | 693 | -0.271 | 0.0916 | No |
| 296 | CA12 |  |  | 698 | -0.274 | 0.0848 | No |
| 297 | CHD7 |  |  | 706 | -0.284 | 0.0716 | No |
| 298 | MMP16 |  |  | 707 | -0.285 | 0.0737 | No |
| 299 | MYO10 |  |  | 710 | -0.305 | 0.0715 | No |
| 300 | ZEB1 |  |  | 717 | -0.317 | 0.0607 | No |
| 301 | PRDX1 |  |  | 719 | -0.326 | 0.0609 | No |
| 302 | HES6 |  |  | 752 | -0.523 | -0.0054 | No |
| 303 | RPS24 |  |  | 757 | -0.595 | -0.0098 | No |
| 304 | FABP5 |  |  | 759 | -1.641 | 0.0000 | No |
Table: GSEA details [plain text format]

  

Fig 2: RICHARDS\_NOWAKOWSKI\_SCIENCE\_2017\_IPC\_DIV2\_UPREG: Random ES distribution      
 Gene set null distribution of ES for **RICHARDS\_NOWAKOWSKI\_SCIENCE\_2017\_IPC\_DIV2\_UPREG**
